# TB-IRIS among HIV patients in an urban private medical clinic, Malawi

**DOI:** 10.1186/1471-2334-14-S2-P41

**Published:** 2014-05-23

**Authors:** Sekeleghe Kayuni

**Affiliations:** 1 MASM Medi Clinics Limited, Blantyre, Malawi

## Introduction

Malawi is one of the countries in sub-Saharan Africa, adversely affected by HIV/AIDS. At least 40% of eligible people are on ART and most of them are initiated ART at very low CD4+ count. Kanjedza Medi Clinic attends to over 5,000 patients monthly and runs an ART clinic of 1,253 clients, with 280 clients managed every month.

We looked at HIV infected patients presenting with symptoms and conditions suggestive of Immune Reconstitution Inflammatory Syndrome (IRIS) and discuss challenges faced in diagnosis and managing them.

## Materials and methods

Case notes of patients who visited our clinic between June 2013 and November 2013 and suspected to have IRIS were accessed and reviewed. The patients presented in the clinic with worsening symptoms at least a week after ART initiation. Screening and diagnosis techniques of possible Opportunistic infections before or at ART initiation were reviewed as well as management of IRIS.

## Results

Five case notes of patients suspected to have IRIS were accessed. 3 were male patients. All the patients had low CD4+ count (< 200c/µl) and suspected Tuberculosis IRIS. 3 were diagnosed with TB through Gene-Xpert testing (none had Rifampicin resistance TB) and 2 on radiological findings (chest x-ray and abdominal ultrasonography).

Two had ART-associated TB IRIS and 3 had unmasking TB-IRIS. 1 new HIV-reactive patient with CD count < 50c/µl, was negative for TB during screening before ART initiation. However, she developed TB-IRIS within 2 weeks of starting ART.

Laboratory diagnosis and TB treatment were done at distant district and tertiary public hospitals due to limited resources and equipment available within and closer to the clinic. 1 died within 1 month of starting TB treatment and IRIS management, due to suspected hepatic complications.

## Conclusion

Predicting and diagnosing IRIS remains a challenge in developing countries, adversely affected by HIV/AIDS. With most patients having late HIV diagnosis and starting ART at lower CD4+ count, there is need to aggressively screen Opportunistic infections and manage possible IRIS.

Better, low-cost diagnostic options needs to be developed and available in limited-resource areas.

